# One‐Year Trajectory of Pain, Function, and Health‐Related Quality of Life in Patients With Plantar Fasciopathy

**DOI:** 10.1002/jfa2.70067

**Published:** 2025-07-16

**Authors:** Cecilie Røe, Marte Heide, Helene L. Søberg, Cathrine Brunborg, Aasne Fenne Hoksrud, Kjersti Myhre, Jens Ivar Brox, Marianne Mørk

**Affiliations:** ^1^ Faculty of Medicine University of Oslo Oslo Norway; ^2^ Department of Physical Medicine and Rehabilitation Oslo University Hospital Oslo Norway; ^3^ Department of Biostatistics, Epidemiology and Health Economics Oslo University Hospital Oslo Norway; ^4^ Norwegian Olympic and Paralympic Committee and Confederation of Sports Oslo Norway

**Keywords:** function, heel pain, long‐term outcome, plantar fasciitis, plantar fasciopathy, rehabilitation

## Abstract

**Introduction:**

Plantar fasciopathy is the most common painful condition in the foot and may imply long‐term pain and functional limitations. The purpose of this study was to assess the time pattern of recovery in pain, function, and health‐related quality of life over 12 months in patients with plantar fasciopathy and assess whether demographic or clinical characteristics could influence these trajectories.

**Methods:**

200 participants referred to specialized care, diagnosed with plantar fasciopathy, and randomly assigned to advice plus customized orthosis or advice plus customized orthosis in combination with radial ESWT, sham‐rESWT, or high‐load exercises were included. Linear mixed model analysis based on demographic information, clinical characteristics, heel pain during activity and at rest the last week, and functioning and health‐related quality at baseline and after 3, 6, and 12 months were conducted.

**Results:**

Pain, function, and health‐related quality of life improved gradually from baseline to 12 months follow‐up with largest improvement within the first 3 months. Bilateral pain was associated with differential trajectories of pain at rest and foot function over time, and a lack of improvement from 6 to 12 months follow‐up.

**Conclusions:**

Different recovery trajectories are important to consider when determining timepoints for follow‐up in scientific studies as well as to individualize the clinical follow‐up.

**Trial Registration:**

ClinicalTrials.gov: NCT03472989

## 1 Introduction

Plantar fasciopathy is the most common painful condition in the foot [1, 2]. The pain is assumed to be derived from the plantar fascia at its insertion on the medial border of calcaneus [3]. Yet, the exact pathophysiological mechanisms involved are debated along with the risk factors for developing this condition [4]. Hence, the term plantar heel pain is also commonly applied for this condition [5]. However, it is well documented that plantar fasciopathy influences functioning substantially and also has consequences for the health‐related quality of life of the patients [6]. Although the condition may be self‐limiting, it is reported that up to 50% of the individuals still suffers from pain 5 years after onset [7]. A wide variety of treatment modalities have been applied without indicating superior approaches [8]. Hence, a best practice guide suggested taping, plantar fascia stretching, advice regarding activities along with orthoses and extracorporeal shock wave therapy (ESWT) for patients with slow recovery [9]. The variations in reported recovery and treatment responses may be related to differences in the patient populations. Patients with foot pain are typically included in studies from medical and physical therapy practices in primary care, from sport clinics, podiatry practices, through advertising, and from specialized hospital units [8]. In a recent study, from a specialized outpatient unit in Norway, advice plus customized foot orthosis and advice plus customized foot orthoses with additional radial extracorporal shock wave therapy (rESWT), sham rESWT, and high‐load strength training exercises were compared [10]. No additive effect of rESWT or high‐load exercise program in recovery of pain, function, or health related quality was found [11]. Sex and bilateral distribution of heel pain has previously documented to predict recovery of heel pain [7] along with duration of pain [12]. In the present cohort, several predictors were identified in the univariate analysis, whereas in the multivariable analysis bilateral distribution of pain was the most consistent predictor for pain and function after 12 months [13].

From a clinical perspective the time pattern of recovery and not only the final outcome is of major importance. It is well established that age influences the mechanical properties of tendons [14] with prolonged time for healing and load tolerance [15]. Subsequently, the time pattern of recovery from pain and disability is likely to be influenced by the age of the patients with plantar fasciopathy. Based on knowledge from painful conditions in general, different trajectories of recovery may also be assumed across other factors like duration of pain [16]. Knowledge of different recovery patterns in the heel pain population is important in order to adjust follow‐up and treatment schedules and to our knowledge not previously explored in patients with plantar fasciopathy.

Hence, the present study aimed to assess the trajectory (time pattern of recovery) for pain, function, and health‐related quality of life over 12 months, whether the trajectories varied across bilateral pain distribution, and demographic and clinical characteristics.

## 2 Methods

### 2.1 Trial Design and Participants

Secondary analysis from a sham‐controlled, observer‐blinded, and partly patient‐blinded RCT with four parallel groups was conducted at the Department of Physical Medicine and Rehabilitation at Oslo University Hospital. The study was performed according to previous published protocol [17] and analyzed according to the statistical analysis plan published in ClinTrials.gov (NCT03472989). Patients were enrolled between March 23, 2018, and January 28, 2022. The study was approved by the Regional Committee for Medical and Health Research Ethics Southeast Norway (2017/1325).

Patients between 18 and 70 years referred from local general practitioners to the outpatient clinic with heel pain, were eligible for inclusion. Inclusion criteria were pain with duration over 3 months localized in the proximal insertion of the plantar fascia on the medial calcaneal tuberosity, and tenderness to palpation corresponding to the painful area. The reported pain intensity during activity last week on a numeric rating scale had to be three or more. The participants had to be residents of Norway and understand oral and written Norwegian. The exclusion criteria were treatment with rESWT within the last 3 months, spondyloarthropathy or rheumatoid arthritis, plantar fibromatosis, tarsal tunnel syndrome, polyneuropathy, previous surgery with remaining osteosynthesis material in the foot or ankle, and contraindications for rESWT (use of anticoagulant drugs, pregnancy, bleeding disorders, epilepsy, or pacemaker).

### 2.2 Participant Involvement

Patients were involved in validation of the outcome assessment foot function index‐revised short form (FFI‐RS). The service‐user panel at the Research Centre for Habilitation and Rehabilitation Models & Services (CHARM), University of Oslo discussed and commented on the results from the RCT.

### 2.3 Treatment and Follow‐Up

Participants were randomized to four treatment groups advice plus customized orthosis only and advice plus customized orthosis combined with rESWT, sham‐rESWT, or heavy slow exercises, respectively. At the first visit, one of three physicians performed a clinical examination and ultrasound scan of each participant. The patients completed questionnaires about sociodemographic and clinical factors and outcomes. A physiotherapist orally communicated the standardized information and advice based on usual care in the department. The participants also received a written hand‐out containing the information. The patients were then referred to an orthopedic technician from certified prothetist/orthotist (CPO), who performed a 3D scan of the foot to prepare the customized foot orthoses. The orthoses were made of a semirigid material named Comfort‐Line (Paromed GMBH, Neubeuern, Germany). The patients received the orthoses after about 3–4 weeks. The patients were provided with instructions to use the orthosis as much as possible and were offered one follow‐up if the orthosis needed further customization. Details of the treatment in the four arms are reported in the protocol [17] and in the reported primary outcome analysis from the RCT [11].

### 2.4 Outcome Measurements

The outcome measurements were pain during the last week measured on a numeric rating scale (NRS), FFI‐RS, and RAND‐12. NRS is an 11‐point numeric scale ranging from 0 “no pain” to 10 “worst imaginable pain” [18], and the participants were asked to rate their heel pain during activity (PainA) and during rest (PainR). In the case of bilateral plantar fasciopathy, the participants were asked to register the most painful heel (right or left), and the same side was assessed at baseline and subsequent follow‐ups.

FFI‐RS is a region‐specific PROM and consists of 34 questions with five subscales: pain, stiffness, difficulty, activity limitations, and social issues [19]. The total score ranges from 0 to 100, where lower score indicate better foot health. We used the translated and validated Norwegian version of FFI‐RS [20]. RAND‐12 Norwegian version [21] is based on the 12‐item short form health survey (SF‐12) v1, a generic patient‐reported outcome measurement (PROM) measuring health‐related quality of life [22]. It consists of 12 questions and generates two scores: mental component summary (MCS12) and physical component summary (PCS12). The scores range from 0 to 100 where higher scores indicate better health.

### 2.5 Statistical Analyses

Descriptive statistics for demographic and clinical information and outcome measures at all follow‐up timepoints were calculated. Within group effect sizes for the outcome measurements were calculated as the difference from baseline to 12 months divided by the pooled standard deviation (Cohens' *d*), with changes above 0.5 medium and above 0.8 large [23]. The missing values were handled by the maximum likelihood estimations of the linear mixed effect models. Since linear mixed effect models did not show any differences between the intervention arms, the linear trajectories over one year (for PainA and PainR, FFI‐RS, PCS12, and MCS12) were examined for the total population [11]. Age, gender (female/male), marital status (married/single), education (≤ 12 years/> 12 years), work status (working/not working), BMI, smoking (yes/no), duration of pain (dichotomized ≤ 12 mnd/> 12 mnd) and pain distribution (unilateral/bilateral), physical activity level, plantar fascia thickness, and number of heel raises were potential predictors. Independent variables were checked for correlation (Spearman's rho). None of the variables correlated above 0.7. Hence, the variables were all entered simultaneously as fixed effects into the first round of linear mixed effect models. All variables were centered or given a reference point of 0 before being entered into the analysis. The trajectories over baseline, 3‐, 6‐, and 12‐months follow‐up were calculated for each of the dependent variables; PainA, PainR, FFI‐RS, PCS12, and MCS12. Main effects would indicate that scores of the dependent variable vary as a function of the predictor variables. A second round of linear mixed effect models were run including significant predictors and their interaction by time. Interaction by time was included for one predictor at a time. All mixed effect models were checked for normal distribution of the residuals and homogeneity of variance. All final mixed effect models were checked for potential improved fit applying quadratic time interactions. Significance level of *p* < 0.05 was adopted. IBM SPSS Statistics v 28.0 was used for the analyses.

## 3 Results

### 3.1 Patient Population

**FIGURE 1:**
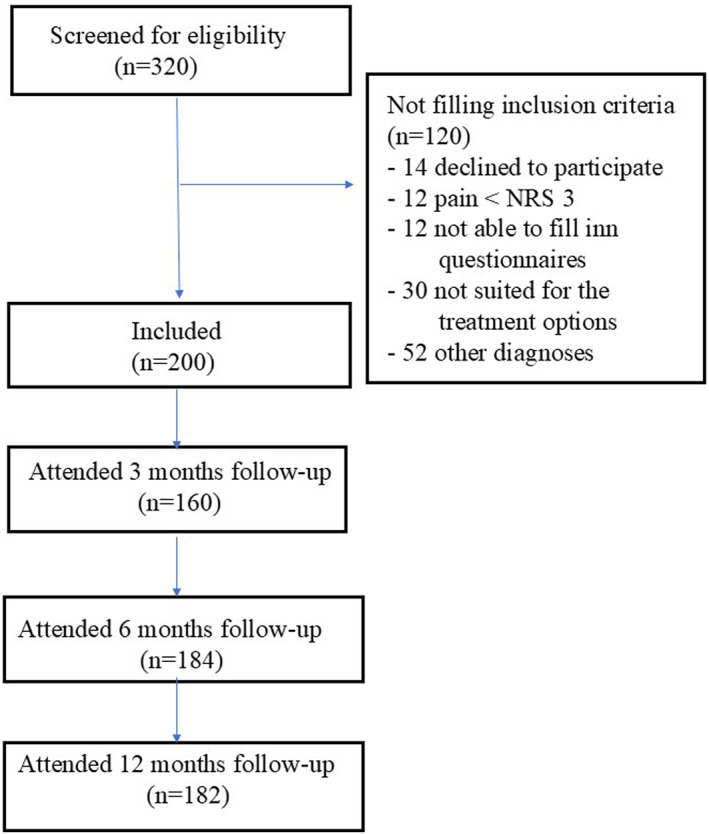
Inclusion and follow‐up of participants.

Of 320 patients referred to the clinic with heel pain, 120 did not meet the inclusion criteria or declined to participate (Figure 1). Hence, 200 were included of which 160 to 182 attended the follow‐ups at 3, 6, and 12 months (Figure 1). Baseline characteristics of the participants are given in Table 1.

**TABLE 1:** Demographic and clinical characteristics of the 200 included participants at baseline.

Baseline characteristics (*n* = 200)	
Age in years^a^	44.8 (10.8)
Sex, female^b^	163 (81.5%)
BMI^a^	28.7 (4.9)
Marital status	
Married/cohabitant	132 (66%)
Single	68 (34%)
Education^b^	
≤ 12 years	72 (36%)
12 years, college/university	128 (64%)
Work status^b^	
Not working	24 (12%)
Working/studying	176 (88%)
Duration in heel pain^b^	
3–6 months	58 (29%)
6–12 months	57 (28.5%)
12–24 months	35 (17.5%)
> 24 months	50 (25%)
Physical activity level^b^ (*n* = 196)	
Sedentary	29 (14.8%)
Walking/biking > 4 h week	122 (62.2%)
Recreational sport, competition	45 (23%)
Smoking, no^b^ (*n* = 197),	174 (88.3%)
Bilateral/unilateral heel pain^b^	76 (38%)/124 (62%)
Heel rises, repetitions one leg^a^	16.5 (9.43)
Plantar fascia thickness mm^a^	5.3 (1.36)

^a^ Values are mean (standard deviation).

^b^ Values are frequency and percentages (%), number of participants (*n*), standard deviation (SD), body mass index kg/m^2^ (BMI), millimeters (mm).

### 3.2 Trajectories of Recovery

**FIGURE 2:**
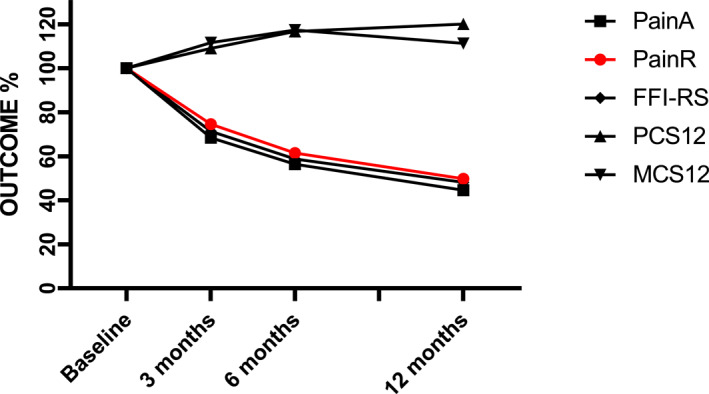
Pain during activity (PainA) and rest (PainR), foot function, that is, foot function index revised short form (FFI‐RS) and health‐related quality of life, that is, the physical component summary (PCS12) and mental component summary (MCS12) from baseline to 12 months follow‐up. Baseline value set to 100% for all outcomes and follow‐up values given in % of the respective outcome value at baseline. (Increase in PCS12 and MCS12 indicate improved quality of life, whereas decrease in FFI‐RS indicates improved function.)

Pain during activity and rest declined significantly from baseline to 12 months follow‐up, foot functioning improved and so did both the physical and mental components of health‐related quality of life (*p* < 0.001) (Table 2). The within group effect sizes from baseline to 12 months were 1.56 and 0.78 for PainA and PainR, respectively. The effect size was 1.18 for FFI‐RS and 0.78 and 0.41 for PCS and MCS, respectively. The percentage of participants with values one standard deviation below the general population mean fell from 84% to 53% for PCS and from 39% to 30% for MCS. The trajectories for PainA, PainR, FFI‐RS PCS12, and MCS12 were quite similar (Figure 2).

**TABLE 2:** Mean (SD) level of pain during activity PainA and rest (PainR), foot function (FFI‐RS), and physical and mental component summary (PCS and MCS) from baseline to 12 months follow‐up.

	Baseline	3 months	6 months	12 months
PainA	6.31 (1.91)	4.32 (2.55)*	3.56 (2.63)*	2.81 (2.59)*
PainR	3.74 (2.41)	2.79 (2.38)*	2.30 (2.34)*	1.86 (2.40)*
FFI‐RS	45.36 (18.06)	32.38 (22.27)*	26.64 (22.31)*	21.84 (21.75)*
PCS12	39.30 (9.45)	42.85 (11.00)*	45.88 (11.27)*	47.20 (10.88)*
MCS12	42.29 (10.71)	43.89 (12.13)*	46.12 (12.25)*	47.09 (12.09)*

* *p* < 0.001 compared to baseline.

In the first round of linear mixed effect analyses we explored whether scores of the dependent variable varied across the various predictors. For each dependent variable, significant predictors (Table 3) were determined and included in the second round of mixed effect models.

**TABLE 3:** Linear mixed effect models exploring the predictors of pain during activity (PainA), pain during rest (PainR), foot function index revised short form (FFI‐RS), and the physical and mental component summary of RAND‐12 (PCS12 and MCS12) over the four time points; baseline, 3, 6, and 12 months follow‐up.

	PainA		PainR		FFI‐RS		PCS12		MCS12	
Predictors	Beta (SE)	*p*	Beta (SE)	*p*	Beta (SE)	*p*	Beta (SE)	*p*	Beta (SE)	*p*
Intercept	**6.53 (0.42)**	**< 0.01**	**4.05 (0.41)**	**< 0.01**	**53.72 (3.55)**	**< 0.01**	**32.45 (1.81)**	**< 0.01**	**34.44 (2.01)**	**< 0.01**
Time	**−1.14 (0.08)**	**< 0.01**	**−0.60 (0.07)**	**< 0.01**	**−7.69 (0.64)**	**< 0.01**	**2.60 (0.33)**	**< 0.01**	**1.56 (0.36)**	**< 0.01**
Marital status	−0.19 (0.19)	0.31	−0.30 (0.18)	0.11	−1.88 (1.60)	0.24	−0.30 (0.82)	0.72	−1.36 (0.91)	0.13
Sex	−0.06 (0.24)	0.81	0.16 (0.23)	0.47	2.50 (1.99)	0.21	−0.25 (1.01)	0.80	0.41 (1.12)	0.72
Age	0.01 (0.01)	0.74	−0.01 (0.01)	0.58	**0.16 (0.08)**	**0.04**	0.02 (0.04)	0.63	**0.17 (0.04)**	**< 0.01**
Smoke	0.22 (0.28)	0.42	0.50 (0.27)	0.06	1.64 (2.36)	0.49	−0.72 (1.21)	0.55	−1.68 (1.35)	0.21
Work	−**0.91 (0.28)**	**< 0.01**	**−0.75 (0.27)**	**< 0.01**	**−12.21 (2.35)**	**< 0.01**	**6.29 (1.21)**	**< 0.01**	**7.26 (1.34)**	**< 0.01**
Education	−**0.43 (0.19)**	**0.03**	**−0.88 (0.19)**	**< 0.01**	**−6.11 (1.63)**	**< 0.01**	**−2.53 (0.83)**	**< 0.01**	1.57 (0.92)	0.09
BMI	−0.03 (0.02)	0.13	**−0.05 (0.02)**	**< 0.01**	−0.09 (0.17)	0.60	0.06 (0.09)	0.49	0.05 (0.10)	0.62
Bilateral pain	**0.61 (0.20**)	**< 0.01**	**0.86 (0.20)**	**< 0.01**	**5.12 (1.70)**	**< 0.01**	**2.29 (0.87)**	**< 0.01**	**−2.26 (0.96)**	**0.02**
Duration of pain	**0.39 (0.19)**	**0.04**	**0.36 (0.18)**	**0.04**	2.30 (1.57)	0.14	−1.21 (0.81)	0.13	−0.51 (0.90)	0.57
PF thickness	0.001 (0.08)	0.95	0.01 (0.08)	0.99	0.32 (0.67)	0.63	−0.22 (0.34)	0.51	−0.20 (0.38)	0.59
Number of heel rises	−**0.06 (0.01**)	**< 0.01**	**−0.04 (0.01)**	**< 0.01**	**−0.55 (0.09)**	**< 0.01**	**0.25 (0.05)**	**< 0.01**	**0.24 (0.05)**	**< 0.01**
Physical activity	0.17 (0.13)	0.19	0.15 (0.12)	0.22	−0.45 (1.06)	0.67	**1.83 (0.54)**	**< 0.01**	**1.73 (0.60)**	**< 0.01**

*Note:* Predictors chosen for the interaction analysis in bold.

Not working, lower education, longer duration of pain, lower number of heel rises along with bilateral pain distribution were significantly associated with higher PainA (Table 3). However, statistically significant differences in the trajectories over time was not documented for any of the predictors.

**FIGURE 3:**
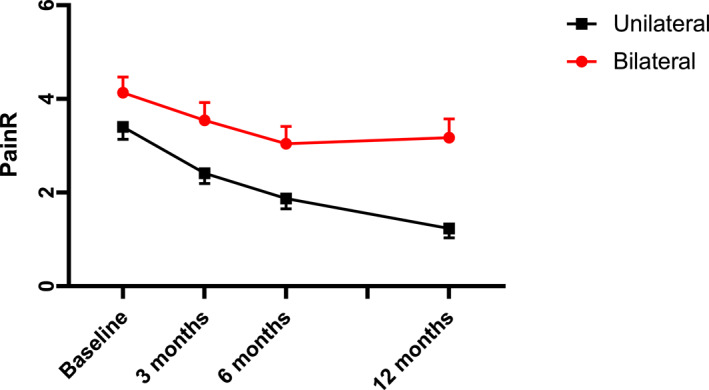
Pain during rest (PainR) (upper panel) and foot function (FFI‐RS) (lower panel) from baseline to 12 months follow‐up for patients with unilateral and bilateral pain distribution. Mean (SE) given.

Not working, lower education, longer duration of pain, lower number of heel rises along with lower BMI and bilateral pain were statistically significant predictors for higher level of PainR (Table 3). In the second round of linear mixed effect models bilateral pain was associated with different trajectories over time compared to participants with unilateral pain (Table 4, Figure 3). The overall model explained 18% of the within subject variance and 54% of the between subject variances.

**TABLE 4:** Second round of linear mixed effect models including significant predictors and significant predictor × time interactions for pain during activity (PainR) and foot function (foot function index revised short form [FFI‐RS]) over 12 months.

	PainR				FFI‐RS			
Predictors	Beta (SE)	95% CI		*p*	Beta (SE)	95% CI		*p*
		Lower	upper			Lower	upper	
Intercept	4.59 (0.44)	3.72	5.45	< 0.01	56.41 (3.88)	48.77	64.06	< 0.01
Time	−0.70 (0.07)	−0.84	−0.56	< 0.01	−8.49 (0.51)	−9.49	−7.49	< 0.01
Age					0.20 (0.11)	−0.03	0.42	0.08
Work	−0.86 (0.38)	−1.61	−0.10	0.03	−11.33 (3.56)	−18.35	−4.31	< 0.01
Education	−0.84 (0.26)	−1.35	−0.32	< 0.01	−6.63 (2.43)	−11.42	−1.84	< 0.01
BMI	−0.06 (0.03)	−0.11	−0.00	0.04				
Number of heel rises	−0.04 (0.01)	−0.07	−0.01	0.01	−0.58 (0.13)	−0.83	−0.33	< 0.01
Bilateral pain	0.49 (0.31)	−0.11	1.10	0.11	2.48 (2.71)	−2.86	7.81	0.36
Time × bilateral pain	**0.25 (0.12)**	**0.02**	**0.48**	**0.03**	**2.45 (0.83)**	**0.82**	**4.08**	**< 0.01**

Based on the first round of linear mixed effect analyses, lower age, higher education, working, and having unilateral pain along with higher number of heel rises were significant predictors of lower FFI‐RS (better foot function) (Table 3). Different trajectories over time were predicted by bilateral pain (Table 4, Figure 3). The overall model including all significant predictors and the quadratic time interaction of bilateral pain explained 31% of the within subjects variance and 73% of the between subjects' variance.

Based on the first round of linear mixed effect analyses lower education, not working, bilateral pain, lower number of heel rise, and physical activity level predicted lower levels of physical health (Table 3). None of the predictors interacted with time.

Based on the first round of linear mixed effect analyses lower age, not working, bilateral pain lower number of heel raise, and physical activity was associated with lower level of mental health (Table 3). None of the included predictors interacted with time, indicating associations with level of MCS, but no differences time development.

## 4 Discussion

In the present study, improvement over time was documented for pain, function, and health related quality of life, but the magnitude of improvement varied across outcome measures. Bilateral pain distribution was associated with a different trajectory over time for pain during rest and foot function. This is to our knowledge the first study exploring factors associated with the trajectories of recovery in heel pain.

The present participants all received a thorough examination by a medical doctor after referral to specialized care. They also received information, advice for activities by a physiotherapist, and customized foot orthosis. Some of the participants received additional treatment with rESWT sham rESWT and standardized exercises [10]. Despite more than 40% of the participants having had heel pain for more than a year, their pain, function, and health‐related quality of life improved markedly over the first 3 months of the RCT. Furthermore, improvement continued at 6 as well as at 12 months follow‐up for all outcome measures. A parallel recovery pattern was observed for the different outcome measures over time, but the magnitude of improvement in the outcome measures varied. The changes in pain during activity and foot function were considered large and the change in mental health considered rather small [22, 23]. Mental health was also less affected than physical health in the present population, and close to the reported level in the general population at 12 months follow‐up [24]. The changes in all outcomes would also be considered to be of clinical importance [25, 26].

In a recent review by Guimaraes et al. [27], rather similar short‐term effects of different treatment modalities regarding pain was documented except for rESWT which was considered to have and additional effect. In the present population we did not find any additional short‐ or long‐term effects of rESWT or exercises [11]. This underscores the importance of advice and education (including foot orthosis) to these patients, recognizing that the lack of control for improvement over time without any treatment [10]. The study by Hansen et al. [7] suggests that about 50% of the patients do not recover regarding pain within 5 years, and reported a number of predictors associated with pain recovery. Rasenberg [28] reported that females had larger functional improvement than men over 6 and 12 months when treated with insoles. Health‐related quality of life is also affected in individuals with plantar fasciopathy [29] but to our knowledge the trajectory over time has not been documented.

In the present study, improvement over time were documented for pain, function as well as health related quality of life, but the magnitude of improvement varied across outcome measures. Heide et al. also documented similar recovery across treatment groups [11]. Also, the individual predictors for level of pain, function, and health‐related quality of life over 12 months varied. Gulle et al. [30] reviewed studies that predicted the outcome of pain and concluded that there was large variability of predictive factors across studies. Population heterogeneity and analytical approach may have also contributed to differences between studies that used the same outcome measure. With close to 50% of participants having had pain for at least 1 year, and close to 40% having bilateral pain, we would assume that our patients were at risk for long‐term symptoms [7, 30]. In the present participants we found that work status, uni/bilateral heel pain, and number of heel raises were predictors of the level of all outcomes. Higher education was also associated with less pain, improved function, and better health‐related quality of life (in the physical component). The methodological approach associating factors with the level of pain over time is slightly different from traditional prediction models usually including a selected time point controlling for baseline levels of the outcome. The slightly different predictors at 12 months identified by Mørk et al. [13] in the same population underscores this. In accordance with the existing literature both demographic and clinical factors contribute [7].

The main aim of the present study was to identify factors that differentially impacted the pattern of recovery over time. Bilateral pain distribution was the only factor not only predicting the level of the outcomes but also a different trajectory of improvement in pain during rest and foot function. Slightly less improvement in response to treatment, but also failing to continue this from 6 to 12 months characterized participants with bilateral pain. These results have clear implications for further studies as well as clinical practice. First of all, representing close to 40% of the present cohort, these participants may interfere with effect evaluation of different treatment modalities. They may also need different treatment strategies. It is well documented that patients with widely distributed pain may need more intensive cognitive behavioral treatment approaches [31]. Possibly, patients with bilateral plantar fasciopathy may benefit from such strategies alone or in combination with the more traditional treatment approaches for foot pain. The lack of improvement from 6 to 12 months indicate that participants with bilateral pain are unlikely to recover untreated, and that they may need longer treatment or at least booster sessions. The causes for different trajectories in patients with bilateral pain may also need to be more thoroughly explored.

The findings of this study need to be considered with several limitations in mind as there are several limitations regarding the results of this study. First of all, the patients were included in an RCT, and the results may not be representative for the patient population with plantar fasciopathy in clinical practice. Secondly, we do believe that bilateral foot pain is a clear predictor for poorer recovery as this result is consistent across all outcome measurements. However, the differences in time pattern of recovery were statistically significant for pain and clearly needs to be reproduced in other studies. Thirdly, the drop out at 1 year follow‐up was below 10%. However, 20% of the patients did not attend 3 months follow‐up. Finally, although imputed according to the model requirement, bias may occur if there is non‐random missing. Future studies should reproduce the different time pattern of recovery in patients with bilateral pain, explore risk factors for bilateral pain, and the response to different treatment strategies in this subgroup of patients with plantar fasciopathy.

## 5 Conclusions

Patients with bilateral pain showed improvement in pain at rest and foot function up to 6 months of treatment but there was a lack of improvement from 6 to 12 months. Different recovery trajectories are important when determining timepoints for follow‐up in scientific studies in order to calculate sample size and evaluate treatment effects appropriately. The results also underscore the need to individualize clinical follow‐up.

## Author Contributions

**Cecilie Røe:** conceptualization (contributing), investigation (contributing), methodology (lead), formal analysis (lead), writing – original draft (lead), writing – review and editing (equal). **Marte Heide:** conceptualization (contributing), investigation (lead), data curation (lead), formal analysis (contributing) writing – original draft (contributing), writing – review and editing (equal). **Helene L. Søberg:** conceptualization (equal), formal analysis (contributing) writing – original draft (contributing), writing – review and editing (equal). **Cathrine Brunborg:** methodology (contributing), formal analysis (contributing), writing – review and editing (equal). **Aasne Fenne Hoksrud:** conceptualization (lead) funding acquisition (lead), formal analysis (contributing), writing – review and editing (equal). **Kjersti Myhre:** investigation (contributing), writing – review and editing (equal). **Jens Ivar Brox:** conceptualization (equal), writing – review and editing (equal). **Marianne Mørk:** conceptualization (equal), investigation (lead together with MH), data curation (lead together with MH), formal analysis (contributing), writing – original draft (contributing), writing – review and editing (equal).

## Ethics Statement

This trial was approved by the Regional Committee for Medical and Health Research Ethics Southeast Norway (2017/1325). Written informed consent was obtained from all patients.

## Conflicts of Interest

The authors declare no conflicts of interest.

## Data Availability

Anonymized data used in the current study can be made available from the corresponding author on reasonable request.
